# Quantum Light Emission from GaAs_*x*_P_1–*x*_ Quantum Dots in Wurtzite GaP Nanowires

**DOI:** 10.1021/acsami.6c06019

**Published:** 2026-06-22

**Authors:** Paolo De Vincenzi, Robert Andrei Sorodoc, Akant Sagar Sharma, Mario Roggi, Isabella Santanchè, Leonardo Perrini, Enrico Mugnaioli, Riccardo Rurali, Fabio Beltram, Lucia Sorba, Valentina Zannier, Marta De Luca

**Affiliations:** † Department of Physics, Sapienza University of Rome, P.le A. Moro 5, 00185 Rome, Italy; ‡ NEST Istituto Nanoscienze-CNR and Scuola Normale Superiore, Piazza S. Silvestro 12, 56127 Pisa, Italy; § Department of Earth Sciences, University of Pisa, Via S. Maria 53, 56126 Pisa, Italy; ∥ Institut de Ciència de Materials de Barcelona (ICMAB−CSIC), Campus de Bellaterra, 08193 Bellaterra, Barcelona, Spain

**Keywords:** nanowires, quantum dots, GaAsP, wurtzite GaP, single-photon emitters, microphotoluminescence

## Abstract

Tunable single-photon emitters in nanowires (NWs) are promising building blocks of compact and efficient quantum photonic devices. We report the vapor–liquid–solid growth of wurtzite (WZ) GaAs_*x*_P_1–*x*_ (*x* = 0.7 and 0.9) quantum dots (QDs) embedded in defect-free WZ GaP NWs, showing high crystalline quality and emission in the 630–700 nm wavelength range. Power- and temperature-dependent microphotoluminescence (μ-PL) measurements show well-resolved single excitonic lines with fwhm < 2 meV up to 70 K. Band structure calculations allow effective modeling of the relation between QD size, alloy composition, and QD emission energy. Second-order autocorrelation measurements demonstrate high-purity single-photon emission, with g^(2)^(0) = 0.090 ± 0.001 under pulsed excitation at 5 K, and antibunching also under continuous-wave excitation, from 5 to 40 K. Time-resolved measurements reveal lifetimes shorter than one nanosecond. Our results show that WZ GaAs*_*x*_* P_1–*x*_ QDs in GaP NWs have potential as wavelength-tunable quantum light emitters under relaxed cryogenic conditions.

## Graphical abstract

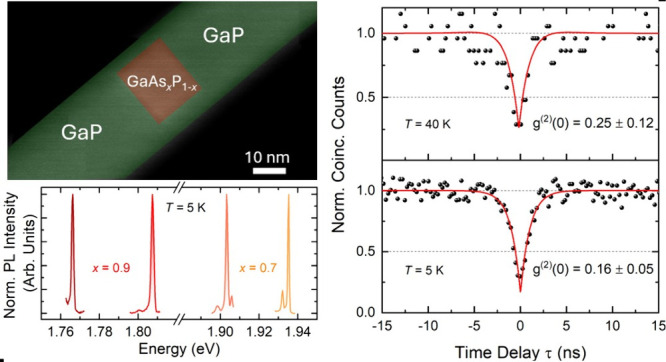

## Introduction

Pure single-photon states are a key element in developing optical quantum technologies, as deterministic generation of quantum light is required for scalable quantum networks and entanglement distribution.^1^ Quantum dots (QDs) are ideal as single-photon sources due to their near-zero dimensionality, which ensures strong carrier confinement, and ease of integration with solid state circuits and devices. III–V semiconductor self-assembled QDs grown in Stranski-Krastanov (SK) mode have demonstrated exceptional optical qualities^2^ and have already found widespread commercial application.^3^ However, SK growth is a strain-driven stochastic process,^4^ for some III–V crystals hardly compatible with Si due to lattice mismatch and often lacking portability since the QD is bound to the growth substrate. Recent advances in bottom-up growth of semiconductor nanowires (NWs) have enabled compositional^5^ or crystal structure^6^ control during synthesis, making III–V NWs with embedded QDs a promising platform to overcome the above-mentioned limitations. The NW geometry naturally allows strain relaxation, enabling epitaxial growth on different substrates of high-quality heterostructures even between highly lattice-mismatched materials,^7,8^ and the growth of crystal phases not achievable in planar geometries.^9^ NWs can also act as intrinsic waveguides as the axial symmetry channels emission along the growth direction, improving extraction efficiency^10,11^ and direct coupling to optical fibers^12,13^ or photonic circuits.^14^

Among III–Vs, GaP gained applicative relevance due to its transparency range (0.6–11 μm)^15^ and minimal lattice mismatch with Si, and, when grown in the NW form, to its pseudodirect bandgap in the wurtzite-phase (WZ) crystal structure in the 2.18–2.25 eV range^16–18^ and to its capability to host thermal-management relevant hetero- and homostructures.^19,20^ Ternary GaAs_*x*_P_1–*x*_ alloys enable bandgap tuning in the green-to-red range of visible light (from 550 to 780 nm), which is a highly attractive spectral window for quantum technologies due to direct compatibility with cost-effective silicon-based single-photon detectors, possibly enabling scalable architectures for quantum computation and communication.^21,22^ Furthermore, this spectral region is also particularly well suited for free-space communications, due to low atmospheric attenuation and the availability of efficient visible-optics platforms,^23^ and for emerging quantum sensing and imaging approaches, including biophotonics and hybrid quantum interfaces.^24^ Prior studies demonstrated antibunched single-photon emission in zincblende (ZB) GaAs QDs in GaAsP NWs up to 160 K at 760 nm with a fwhm of ∼2 meV.^25^ GaAs QDs embedded in GaAsP ZB NWs showed narrow emission lines at 710 nm, fwhm < 10 meV, up to 140 K.^26^ Furthermore, compositional control allowed the growth of GaAsP QDs in GaP NWs in both ZB^27^ and WZ^28^ phases, as well as other quantum-confined architectures such as radial heterostructures and quantum wires.^29,30^

In this work, we demonstrate single-photon emission from WZ phase GaAs*_*x*_* P_1–*x*_ QDs with optimized morphology embedded in defect-free WZ GaP NWs, fabricated by optimized catalyst-assisted vapor–liquid–solid (VLS) growth by chemical beam epitaxy. Power- and temperature-dependent optical measurements revealed intense, and well-resolved, single excitonic lines with 440 μeV line width at 5 K. Electronic band structure calculations allowed us to correlate the effect of quantum confinement and alloy composition with the emission energy, providing a predictive framework for emission tuning. Temporal autocorrelation measurements showed single-photon emission at 5 K (g^(2)^(0) = 0.090 ± 0.001), with subnanosecond lifetime and antibunching maintained up to 40 K and with a realistic possibility to reach 77 K upon nanostructure optimization (through better surface passivation and waveguiding profile). Previously, μ-photoluminescence (μ-PL) measurements on similar WZ GaAs*_*x*_* P_1–*x*_ QDs embedded in defect-free WZ GaP NWs^28^ revealed bright, spatially localized QD-like emission along the nanowire axis, tunable from 650 to 720 nm depending on the incorporated As content, up to ≈70 K. However, no antibunching behavior was reported. In contrast to these earlier studies, the present work introduces a growth-optimization strategy aimed to enhance confinement and suppress structural and surface-related defects,^31,32^ based on a diluted solution of 10 nm Au catalyst colloidal nanoparticles (NP) instead of the previously used 20 nm NPs.^28^ The reduced size leads to thinner NWs with smaller QDs and thus enhanced quantum confinement. This morphological change enables, for the first time, the observation of single-photon emission and provides improved control over emission properties.

**Figure 1:**
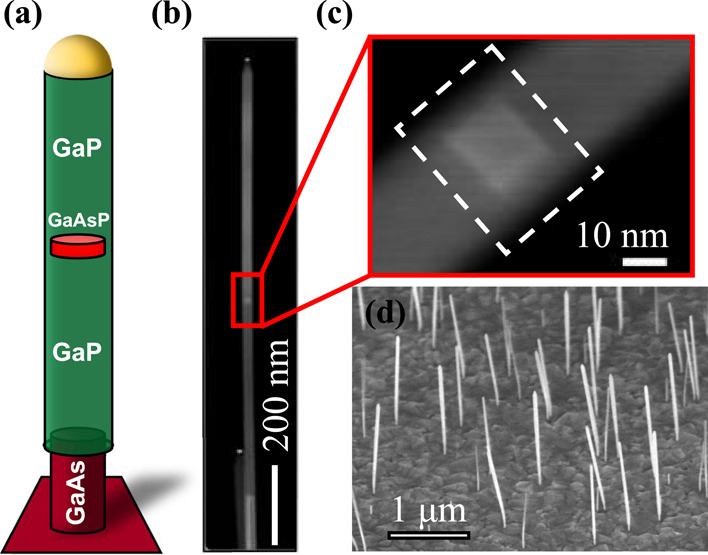
(a) A schematic representation of the WZ GaP/GaAsP QD-NW. The GaAs substrate and stem are shown in dark red, the GaP segments are green, the QD is light red, and the Au NP is yellow. The figure is not to scale. (b) STEM image of a single GaP NW with a clearly visible GaAs_0.7_P_0.3_ QD. In the bottom, a portion of the GaAs stem is visible as well as a thin GaP shell around it. (c) Zoomed STEM image of the GaAs_0.7_P_0.3_ QD, surrounded by a thin GaP shell. (d) 45°-tilted SEM image of the as-grown NW ensemble. Two populations of NWs are visible: thin and short, thick and long NWs.

## Results and Discussion

A schematic representation of the NW structure is shown in Figure 1a, and growth details of the newly optimized procedure are reported in SI1. Structural characterization by scanning transmission electron microscopy (STEM) and electron diffraction confirmed a nearly defect-free WZ crystal structure with sharp interfaces between the GaAs*_*x*_* P_1–*x*_ QD and the GaP barriers. As shown in Figure 1b,c, NWs show no tapering and a thin passivation shell (a few layers-thick) that was previously shown to enhance QD emission.^28^ The GaAs(111)­B substrate imposes the hexagonal symmetry to the epitaxially grown NWs. A ∼300 nm GaAs stem was first grown to promote defect-free axial growth across the GaAs/GaP large lattice mismatch. Subsequently, 1600 ± 200 nm WZ GaP NW segments were obtained. A single GaAs*_*x*_* P_1–*x*_ QD was embedded along the wire axis, with As concentrations of 70 and 90%. Figure 1d shows a 45°-tilted SEM image of the as-grown ensemble of NWs, revealing two main NW populations: thin and short, thick and long wires. Despite the initial narrow size distribution of the Au colloids, the presence of NWs with a broad diameter distribution is commonly observed. When annealing the substrate, multiple nearby colloids merge into one single particle, giving rise to larger NWs. At the same time, individual particles can split into smaller NPs. Therefore, the final NW diameter distribution is larger than the initial NP size distribution, frequently showing multiple peaks.^33^ STEM observations showed that the passivation shell has nearly constant thickness on all wires, regardless of their diameter (in Figure S2 in SI1), allowing to establish a correspondence between NW thickness and QD diameter, the latter mainly depending on the NP diameter. As illustrated by the scatter plot of measured QD height (*h*) versus QD diameter (*d*) in Figure 2a, statistical analysis confirms the presence of two distinct QD populations whose distribution can be described by the Gibbs–Thomson trend: small QDs in thin NWs, ∼ 15% of the total, with *h* = 6 ± 1 nm and *d* = 10 ± 3 nm, and bigger QDs in thicker NWs, with *h* = 14 ± 2 nm and *d* = 22 ± 5 nm. For larger diameters, the distribution shows instead an opposite trend with slightly thinner QDs as the NW diameter increases, due to surface diffusion mechanism dominating in this range.^34^ Smaller QD volumes directly translate into narrower, and blue-shifted emission lines, as will be discussed in the μ-PL results.

**Figure 2:**
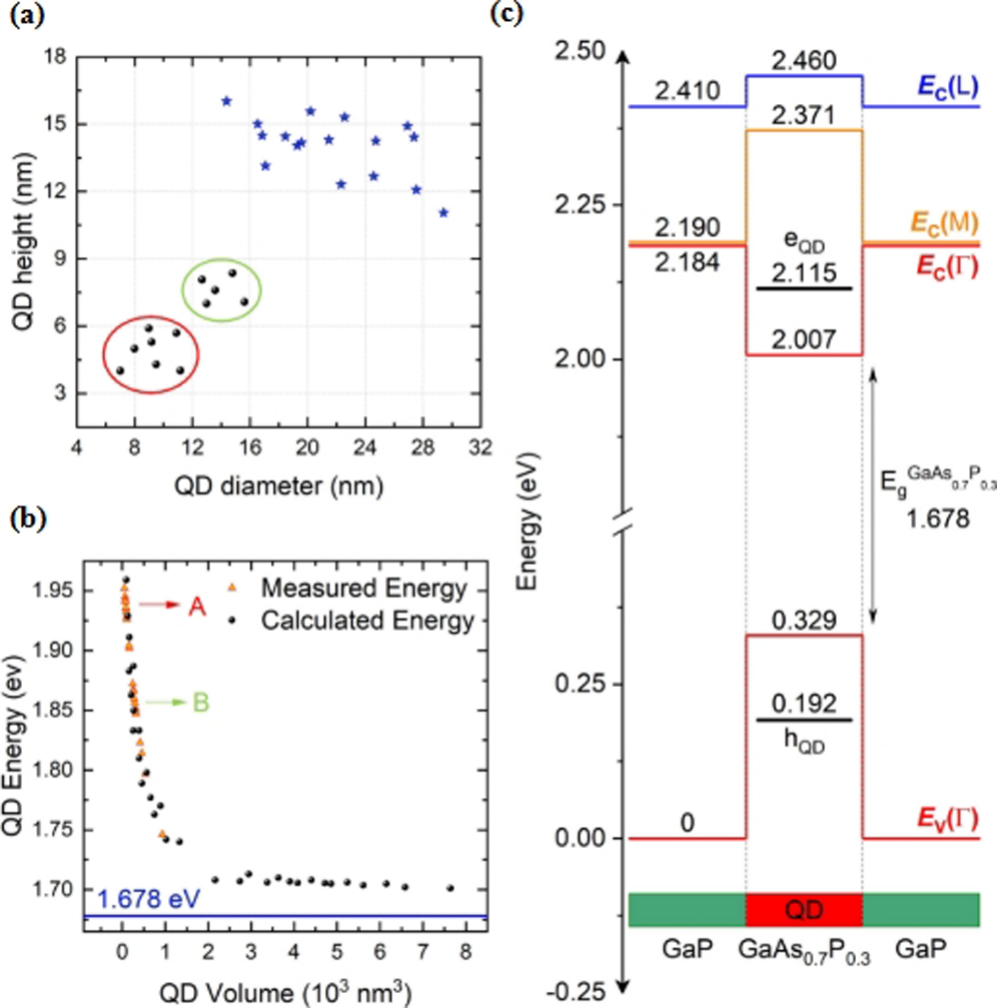
(a) Scatter plot of the QD height (±1 nm) as a function of QD diameter (±2 nm), measured through STEM. Data loosely follow a Gibbs–Thomson distribution. Two populations can be recognized: small QDs in thin NWs (black dots) and bigger QDs in thick NWs (blue stars). Green and red circles highlight the existence of thin and ultrathin NWs, respectively, containing the smallest QDs. (b) QD first transition energy as a function of total volume, calculated using Nextnano software (black dots). QD height and diameter contribute differently to confinement, as shown in SI3. The orange triangles show the peak energies measured in μ-PL (±0.5 meV), for which the volume of each QD (±10% error for QDs in thick NWs and ±30% error for QDs in thin NWs) was determined by comparison with the calculations. A and B highlight the emission of QDs belonging to ultrathin and thin NWs, respectively. The blue line shows the reference, unconfined, bandgap energy of WZ GaAs_0.7_P_0.3_. (c) Band edge diagram, calculated with Nextnano software, of the NW QD heterostructure showing the WZ GaP barriers (in green) and a GaAs_0.7_P_0.3_ QD of group A (in red) with diameter 7 nm and height 4 nm. QD first transition recombination energy is 1.923 eV. All given numbers in the diagram are energies in eV.

However, the substantial variability in the overall confinement volume limits energy homogeneity on a given sample, as seen through the μ-PL characterization of multiple QDs (in SI2): for a fixed As concentration (within ±2.5%), the emission energy varies over a range of ∼150 meV, while line width spans from 0.4 to 2 meV across different emitters. On one hand, this inhomogeneity limits scalability in applications requiring identical and deterministic QD energies, on the other hand, it can be considered as an additional degree of freedom for the tunability, because a fast μ-PL scan over the ensemble can be exploited to select the desired QD energies for a given application. To establish a correlation between the optical signal and the dimensions of these novel WZ QDs, whose band structure is unknown, numerical simulations were performed with the Nextnano software.^35^ The electronic band structures of WZ GaP and GaAs were derived from density functional theory calculations,^36^ from which effective masses at high-symmetry points were extracted. Lattice parameters were measured by three-dimensional electron diffraction (3DED)^37^ in TEM on reference samples of WZ GaP and GaAs NWs with low defect density and negligible strain, obtaining *a* = 3.89 Å, *c* = 6.40 Å and *a* = 4.01 Å, *c* = 6.64 Å, respectively, consistent with previous reports.^28,36^ QD band structure and energy levels were computed by self-consistently solving the 3D effective-mass Schrödinger and Poisson equations, as further explained in the SI1. Figure 2b shows the calculated energy, at 5 K, (black dots) of the QD first transition as a function of the confinement volume, which follows the *E* ∝ *V* ^–2/3^ trend of the particle-in-a-box approximation. Simulations highlight an anisotropic contribution of height and diameter to the confinement: for QDs with comparable volume, reduced height leads to stronger confinement and higher emission energies. The effect can be more easily seen in the scatter plot in SI3. In Figure 2b, experimental data (orange triangles) are plotted as a function of volume. For large QDs, emission is distributed across a plateau at ∼1.70 eV, just above the WZ GaAs_0.7_P_0.3_ bandgap value of 1.678 eV, evidence of a low degree of confinement. This is consistent with the measurements, as narrow and bright emission was not observed at those energies. Below the critical volume of ∼1.5 × 10^3^ nm^3^, a strong-confinement regime is observed as QD dimensions approach the exciton Bohr radius, from Nextnano, leading to more spaced levels at higher emission energy as experimentally observed. The agreement between simulations and μ-PL allowed us to establish a correspondence between QD energy spectrum and size. In fact, two subgroups can be recognized within the population of thinner wires, as highlighted by the green and red circles in Figure 2a, that can be associated with measured QD energies of group B (thin NWs) and A (ultrathin NWs), respectively, in Figure 2b. The band edge diagram of a representative QD of group A, calculated with Nextnano, is reported in Figure 2c. A diameter of 7 nm and height of 4 nm were chosen to match the emission energy of the representative QD with 70% As concentration, on which μ-PL analysis was performed. In the diagram, the zero-energy level is set to the valence-band maximum of WZ GaP at Γ. The GaP barriers exhibit a pseudodirect bandgap with the conduction-band minima at Γ and M separated by ≈6 meV, consistent with previous reports.^16,17,38^ In the QD region, quantum confinement results in discrete levels for electrons and holes, allowing radiative recombination at energies higher than the estimated bandgap of the material. Comparison with the band-edge diagram calculated for a larger-volume QD shows red-shifted levels, confirming the expected confinement dependence, in SI4. Finally, comparing QDs of identical volume but different As% (70 and 90%) confirms the preservation of emission tunability for equal-size QDs, from 1.923 to 1.838 eV, respectively, in SI4. Therefore, band structure calculations indicate that the primary growth-controlled tunability arises from the incorporated As concentration in the GaAs_*x*_P_1–*x*_ alloy, while variations in the QD dimensions provide an additional contribution to the confinement energy and consequently broaden the accessible emission-energy range.

The optical emission properties of a single vertical GaAs_0.7_P_0.3_ QD NW, belonging to group A of the thin NWs, were characterized as a function of excitation power and temperature. Measurements were performed in a backscattering geometry, with laser and PL *k* vectors parallel to the NW growth axis; further experimental details are in SI1 as well as in the Methods section below. Figure 3a shows the μ-PL spectra under nonresonant excitation, with varying impinging power, from 50 nW to 10 μW. At *P*_0_ = 1 μW, the spectrum is dominated by the intense narrow peak, QD_1_, at 1.935 eV. As excitation power increases, the integrated PL intensity of the emission increases linearly, *I*_det_ ∝ (*P*_exc_)^*M*^ with *M*_1_ = 1.08 ± 0.03, see inset of Figure 3b, which is typical for fundamental single neutral excitonic transitions in QDs.^39^ At higher powers, intensity saturation occurs with a sublinear trend, as shown in Figure 3b, indicating QD state-filling without the appearance of higher-energy peaks or barriers emission,^17,40^ as instead observed in similar but bigger dots (*d* > 20 nm, *h* ∼ 12 nm),^28^ evidencing efficient carrier confinement and high crystalline quality. Power-dependent line width broadening was also studied: at low powers, it reaches the limit value of fwhm_0_ ∼ 440 μeV, which is most likely limited by our spectral resolution, and is comparable with the best performing QD NWs in similar materials.^25,26,41,42^ A hybrid model was used for fitting by considering the homogeneous power broadening of two-level systems,^43^ $\mathrm{fwhm}(P)={\mathrm{fwhm}}_{0}\sqrt{1+P/P_{\mathrm{sat}}}$ , and inhomogeneous contributions from charge fluctuations,^44^ fwhm­(*P*) = fwhm_0_ + *bP* ^α^. *b* is a proportionality coefficient depending on QD crystal quality and trap states density while α represents the fwhm broadening power-dependence. The analysis, combined with the absence of emission shift due to laser-induced heating, suggests the predominance of inhomogeneous broadening arising from spectral diffusion attributed to a local Stark effect induced by local electric field fluctuations, which is known to be the dominant light-driven broadening mechanism in QDs.^45,46^ The fwhm saturates with α ≈ 0.5 and at saturation power, *P*_sat_ = 2 μW, has a value of ∼0.9 meV, which is a substantial improvement with respect to the larger QD sample, which had a minimum fwhm of 2 meV.^28^ No biexciton emission was clearly resolved, likely due to a combination of weak built-in polarization-induced electric fields in the WZ heterostructure^47^ and the spectral broadening induced by surface-related charge fluctuations in thin nanowires without optimized passivation shell. In Figure 3a, other less intense features are also distinguishable at higher powers, QD_2_ (1.932 eV), QD_3_ (1.926 eV), QD_4_ (1.928 eV). They are associated with other QD NWs near QD_1_, their optical and spatial characterization is reported in SI5 and SI6. Figure 3c shows temperature-dependent measurements on QD_1_, with multiplication factors showing intensity quenching as the temperature rises.^48^ The emission undergoes a Varshni-like redshift that was fitted with the one-oscillator model,^49–51^ *E*_*g*_(*T*) = *E*_*g*_(0) – *S*⟨ℏω⟩[ *coth* (⟨ℏω⟩/2*k*_*B*_ *T*) – 1]. *E*_*g*_(0) is the band gap at zero temperature, *S* is a dimensionless coupling constant, and ⟨ℏω⟩ is the average phonon energy. QD_1_ peak at 60 K is red-shifted by 55 meV. At 65 K it is not possible to spectrally resolve the QD_1_ line anymore. The integrated intensity was fitted by an Arrhenius-like model,^52^ in Figure 3d, with an extracted activation energy of *E*_1_ = (27 ± 2) meV, evidence of depopulation toward thermally activated nearby defect or surface-related states, leading to emission quenching with increasing temperature, consistent with what was measured in ref 28. No higher-energy peaks are observed as the excitonic peak remains the dominant recombination channel. The inset of Figure 3d shows the line width trend as the temperature increases; at 65 K, fwhm ≈ 1.3 meV. The fwhm broadening was fitted with the standard phononic model Γ­(*T*) = Γ_0_ + γ_*AC*_ *T* + Γ_*LO*_/[ exp (*E*_*LO*_/*k*_B_ *T*) – 1],^53^ where Γ_0_ represents the intrinsic broadening at low *T* due to surface states, dangling bonds and spectral diffusion,^44,46,54,55^ the linear term γ_*AC*_ is the acoustic phonons interaction with thermally activated sidebands,^56^ and the exponential term describes the coupling with longitudinal optical phonons (LO) of energy *E*_*LO*_. Two distinct regimes can be identified: for *T* < 30 K, the fwhm is linearly dependent on temperature, the contribution of acoustic phonons sidebands is weak compared to the intensity of the excitonic transition. For *T* > 30 K, a superlinear increase in the fwhm is observed due to the dominant role of the optical phonons.^57,58^ Fitted data yield a reduced Γ_0_ = (390 ± 20) μeV, which is much greater than the Fourier-limited line width, indicating that the emission is not lifetime-limited but dominated by dephasing processes. The radiative lifetime was extracted from time-resolved photoluminescence measurements that will be discussed later and in SI7. The extracted coefficients, γ_*AC*_ = (6 ± 1) μeV/K, Γ_*LO*_ = (20 ± 10) meV, *E*_*LO*_ = (20 ± 3) meV are compatible with literature values for III–V QD NWs.^59–61^ Such line width dependence with temperature is comparable with, and in some cases better than, performances of QD NWs of similar composition,^25,26,41^ though still higher than the sub-100-μeV line widths showed by the best performing QDs.^62–64^ In Figure 3c, it can also be seen that the QD_1_ relative intensity with QD_2_ changes as temperature increases, due to slightly different dynamics in the two QDs with different confining dimensions and local environment.

**Figure 3:**
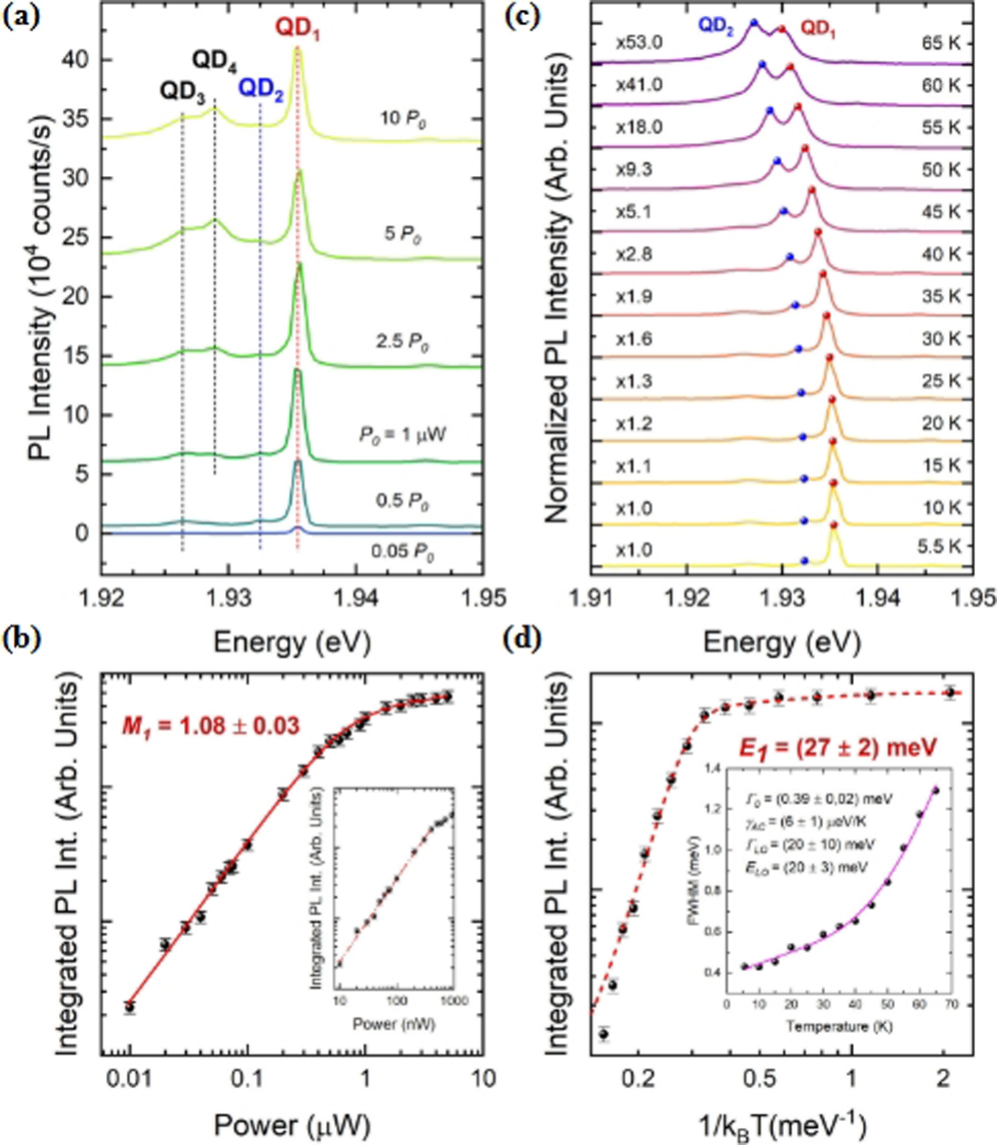
Power- and temperature-dependent μ-PL measurements on a single GaAs_*x*_P_1–*x*_ QD NW with *x* = 70%, belonging to ultrathin (group A) NWs. (a) Stacked power-dependent spectra at 5.5 K of QD single excitonic line QD_1_, red dashed line. Other dashed lines highlight excitonic recombination lines (QD_2_, QD_3_, and QD_4_) from other neighboring QDs. *P*_0_ = 1 μW. (b) Plot of the power-dependent integrated intensity of QD_1_. The red line shows the fit with $I=I_{\mathrm{sat}}\left[\frac{P}{P+P_{N}}\right]$ , where *P*_*N*_ = 0.95 ± 0.07 μW is the laser power at which the intensity is half of *I*_sat_. The inset is a zoom below *P*_0_ to highlight the linear trend before saturation. The dashed red line shows the fit with *I*_det_ ∝ (*P*_exc_)^*M*^ that yields *M*_1_ = 1.08 ± 0.03. (c) Temperature-dependent μ-PL, acquired at *P*_0_, with given intensity normalization factors. Red and blue dots show the thermal redshift of QD_1_ and QD_2_, respectively. (d) Plot of the temperature-dependent integrated intensity of QD_1_ as a function of the reciprocal of the temperature with the Arrhenius fit (dashed red line) and extracted activation energy. The inset shows the variation of the fwhm of QD_1_ with temperature. The data are fitted with the phononic model (purple line), and the extracted parameters are reported.

To characterize the single-photon purity of these novel emitters, second-order correlation measurements, g^(2)^(τ), of QD_1_ were performed in a standard Hanbury–Brown and Twiss setup, exciting the NW in the same configuration as the studies in Figure 3. Emission lines of the different QDs are sufficiently separated in energy to be effectively resolved by the spectrometer, and a narrow exit slit (∼1 nm bandwidth) is used to spectrally isolate the emission of the targeted QD (QD_1_). Furthermore, since each excitonic line originates from different nanowires within the ensemble, as shown in the μ-PL mapping in SI6, any residual contribution from spectral tails of nearby emission is further minimized by the spatial selectivity of the measurement. This combined strategy strongly suppresses spectral crosstalk between adjacent emitters and ensures that the reported g^(2)^(τ) measurements reliably reflect the emission properties of the targeted QD. Results under continuous-wave (CW) excitation at *T* = 5 K are shown in Figure 4a. At low excitation power, a clear antibunching dip is visible with g^(2)^(0) = 0.16 ± 0.05, confirming single-photon emission. As the excitation power increases, the dip becomes shallower, with g^(2)^(0) = 0.32 ± 0.03 at saturation power (*P*_sat_ ≈ 2 μW). Above saturation, g^(2)^(0) approaches and eventually exceeds 0.5, consistent with re-excitation processes that increase multiphoton probability. This power-dependent behavior can be described within the rate-equation framework of Regelman et al.:^65^ *g* ^(2)^(τ) represents the probability of finding the QD occupied by a single exciton at time delay τ after emission of a photon that left it empty. At low pump powers, the repopulation time is long compared to the exciton lifetime, leading to *g* ^(2)^(0) → 0; increasing the pump power accelerates repopulation, narrowing the antibunching notch and raising the probability of simultaneous photon detections. Assuming that multiexciton recombinations’ emit into distinct spectral lines and that, at low powers, the probability of multiple exciton occupancy is negligible (as supported by our μ-PL analysis), the autocorrelation intensity can be modeled by the simplified two-level rate equation: $g^{\left(2\right)}(\tau )=1-\exp\left[-\tau \left(\frac{1}{\tau_{1}}+G\right)\right]$ , where *G* is the CW photogeneration rate of e–h pairs and τ_1_ the radiative lifetime of QD_1_. By using τ_1_ independently measured from time-resolved PL (TRPL) performed in the same experimental conditions, good agreement with the experimental data was achieved, quantitatively reproducing the transition from antibunching at low power to multiphoton emission, further details are in SI7.

**Figure 4:**
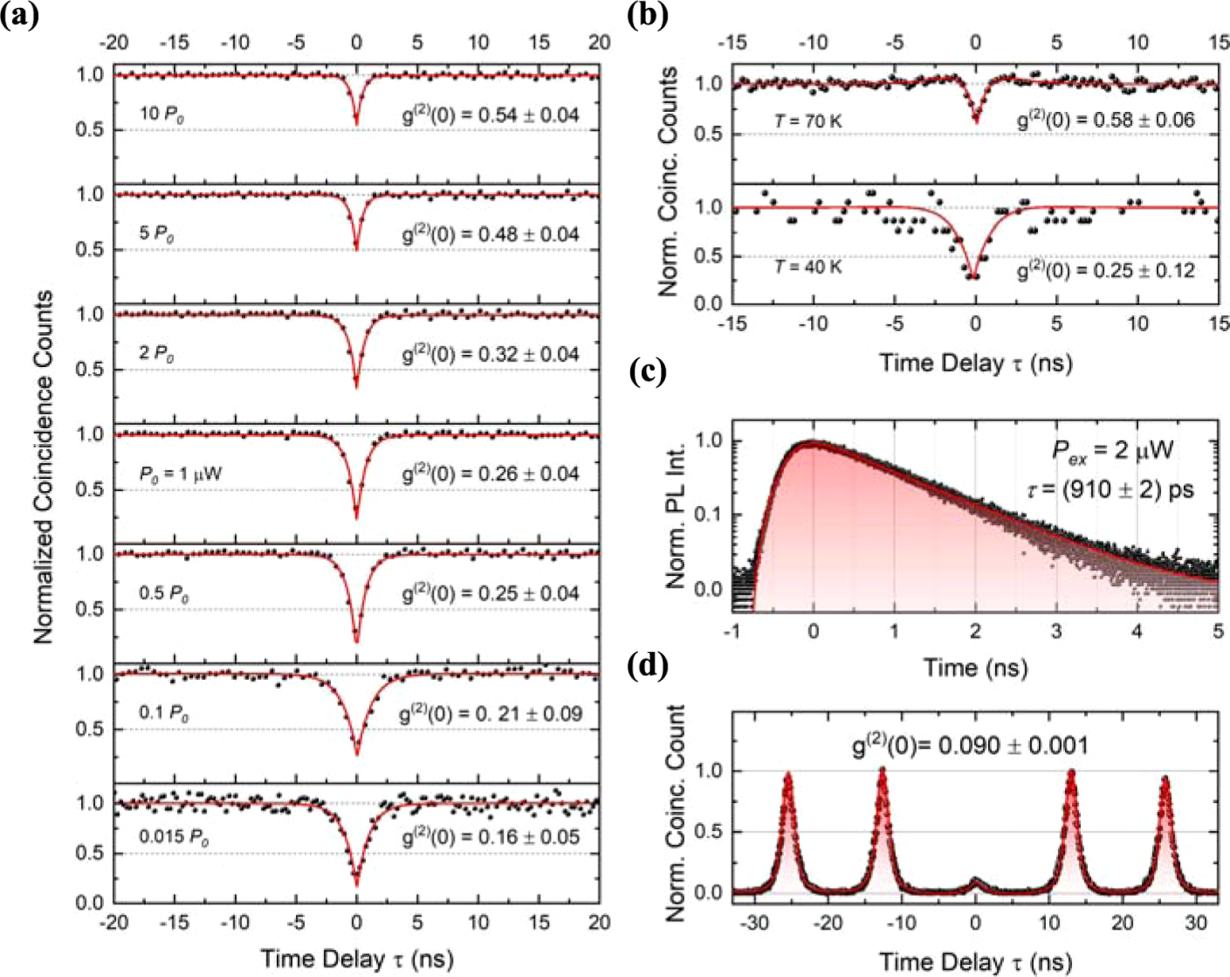
Second-order autocorrelation, g^(2)^(τ), and time-resolved measurements (black dots) on the single GaAs_0.7_P_0.3_ QD NW also measured in Figure 3. Fitting functions are shown with continuous red lines. (a) Temporal intensity autocorrelation function at 5 K of the QD_1_ spectral line under CW excitation for various powers. *P*_0_ = 1 μW. (b) Temporal intensity autocorrelation function of the QD_1_ spectral line at 40 and 70 K, at *P*_0_ CW excitation power. (c) Time-resolved PL on the QD_1_ spectral line at 5 K and 2*P*_0_ pulsed excitation power. Data were fitted with a single-exponential decay function taking into account the system response and background. (d) g^(2)^(τ) measurements of the QD_1_ spectral line under pulsed excitation at 5 K. Repetition rate ∼ 80 MHz.

Figure 4c shows the 5 K TRPL measurement of X_A_ spectral line with *P* = 2 μW pulsed excitation power. The fit (red line) yields τ_1_ = (910 ± 2) ps. TRPL data were fitted by the convolution of the instrumental response function of the setup and the *e* ^–*t*/τ_1_^ function.

To suppress repumping of the QD, *g* ^(2)^(τ) measurements under pulsed excitation were performed by choosing an excitation period much longer than the exciton lifetime (repetition rate ∼80 MHz, corresponding to a pumping period of ∼12.5 ns). Thus, re-excitation processes that limit the CW performance were strongly suppressed, with a Lorentzian fit yielding *g* ^(2)^(0) = 0.090 ± 0.001, red line in Figure 4d, which is in close agreement with the raw value of *g* ^(2)^(0) = 0.101 ± 0.004, obtained by integrating the coincidence counts histogram within a ± 6.25 ns interval around zero delay and comparing the resulting area to those of the nearest normalized adjacent peaks. This confirms that, once repumping is minimized, the QD behaves as a high-purity single-photon source, in agreement with expectations for a two-level emitter driven by a pulsed excitation scheme, and shows the ability to prepare a trigger controlled well-defined single-exciton state, which is essential for single-photon quantum photonic applications. Surface states, background signal, and nonresonant excitation limit the minimum achievable value, but purity can be improved by optimizing the passivation shell or excite resonantly the QD. The brightness of the emitter was evaluated in terms of photon extraction efficiency by correcting the detected count rate (∼44 kcounts/s) for the total optical transmission of the setup (from source to APD), 8.5% ± 1%, and for the detector quantum efficiency (70%). Under pulsed excitation at saturation power (80 MHz repetition rate), this yields a photon extraction efficiency of approximately 0.9% ± 0.2% per excitation pulse, further details are in SI8. Lastly, temperature-dependent *g* ^(2)^(τ) measurements performed below saturation power, in Figure 4b, showed degradation of photon purity, *g* ^(2)^(0)^40K^ = 0.25 ± 0.12 and *g* ^(2)^(0)^70K^ = 0.58 ± 0.06, attributed to two main factors: residual background emission from a neighboring QD (QD_2_) whose spectral tail cannot be fully suppressed by spectral filtering centered on QD_1_ wavelength anymore due to progressive thermally induced broadening of both emission lines (as can also be seen in Figure 3c) and carrier delocalization, as evidenced by the PL analysis, which reduces confinement and increases the probability of multiphoton events. Nonetheless, these results establish a promising benchmark for single-photon operation under elevated cryogenic temperatures, with good current performance maintained up to ∼40 K and a realistic pathway toward operation approaching liquid-nitrogen temperature (∼77 K) through device optimization.

## Conclusions

In conclusion, smaller GaAs_*x*_P_1–*x*_ (*x* = 0.7, 0.9) QDs in WZ GaP NWs were grown through optimized catalyst-assisted VLS growth by chemical beam epitaxy from 10 nm Au NPs, and, for the first time in this material platform, single-photon emission with clear antibunching was demonstrated. By combining μ-PL spectroscopy and band structure calculations, a direct correspondence between emission energy and confinement volume was established. Second-order photon correlation measurements demonstrated pure single-photon emission from GaAs_0.7_P_0.3_ QDs, and TRPL showed subnanosecond radiative lifetime. Power-driven photon bunching at 5 K was properly modeled. Temperature-dependent measurements further showed antibunching up to 40 K, indicating good thermal stability of the emission. The observed performance is enabled by the optimized growth conditions, which reduce nanowire diameter and enhance quantum confinement, highlighting the importance of catalyst size engineering for emission control. Further growth optimization is required to reach a higher control over the QD dimension (and thus emission energy) homogeneity, to realize waveguiding shells that can enhance brightness and collection efficiency. This last improvement could also lead to robust single-photon emission at higher temperatures. Overall, these results promote a novel QD-in-NW system to the toolbox of III–V NW-based single-photon emitters and highlight the potential of GaAsP/GaP WZ QD NWs as growth-tunable single-photon sources for integrated photonic devices in the visible spectral range.

## Methods

Temperature-dependent μ-PL measurements were performed in a closed-cycle helium cryostat. As CW excitation source, a 532 nm solid-state laser was used. For TRPL and autocorrelation measurements, a tunable supercontinuum light source with filter set at 525 nm wavelength was employed. All the μ-PL measurements were performed in backscattering geometry with a 100× objective. Signal was dispersed by a 300 grooves/mm grating and detected by a CCD, providing a spectral resolution of approximately 0.4 meV. TRPL and autocorrelation measurements were performed with different single-photon avalanche photodiodes. More details are provided in the SI1. The SI1 also provides details on growth process, structural characterization, and band structure calculations.

## Supplementary Material



## Notes

SI1: Experimental and band structure calculation methods and shell thickness estimation; SI2: additional optical spectroscopy data on multiple quantum dots; SI3: confinement as a function of dot diameter and height; SI4: band edge diagrams of quantum dots of different size and As%; SI5: power- and temperature-dependent photoluminescence characterization of QD_2_ and QD_3_; SI6: dot emission spatial localization; SI7: power- and temperature-dependent *g* ^(2)^(0) and time-resolved photoluminescence fits; and SI8: estimation of efficiency (PDF)
