# Supplementary material for: Quantum Light Emission from GaAs x P1–x Quantum Dots in Wurtzite GaP Nanowires
Source: ACS Appl Mater Interfaces. 2026 Jun 22;18(25):35624–32. doi: 10.1021/acsami.6c06019 (PMC13339018; doi:10.1021/acsami.6c06019)
Supplement: Supplementary file 1 [file am6c06019_si_001.pdf]

Supporting Information

Quantum light emission from $GaAs_xP_{1-x}$ quantum dots in wurtzite GaP nanowires

*Paolo De Vincenzi[1], Robert Andrei Sorodoc[2], Akant Sagar Sharma[1], Mario Roggi[1], Isabella Santanchè[1], Leonardo Perrini[1], Enrico Mugnaioli[3], Riccardo Rurali[4], Fabio Beltram[2], Lucia Sorba[2], Valentina Zannier[2] and Marta De Luca[1]**

[1] Department of Physics, Sapienza University of Rome, P.le A. Moro 5, 00185 Rome, Italy

[2] NEST Istituto Nanoscienze-CNR and Scuola Normale Superiore, Piazza S. Silvestro 12, 56127 Pisa, Italy.

[3] Department of Earth Sciences, University of Pisa, Via S. Maria 53, 56126 Pisa, Italy

[4] Institut de Ciència de Materials de Barcelona (ICMAB−CSIC), Campus de Bellaterra, 08193 Bellaterra, Barcelona, Spain.

**email: marta.deluca@uniroma1.it*

Contents

SI1. Experimental and band structure calculation methods, and shell thickness estimation

SI2. Additional optical spectroscopy data on multiple quantum dots

SI3. Confinement as a function of dot diameter and height

SI4. Band edge diagrams of quantum dots of different size and As%

SI5. Power- and temperature-dependent photoluminescence characterization of QD_2 and QD_3

SI6. Dot emission spatial localization

SI7. Power- and temperature-dependent $g^{(2)}(0)$ and time-resolved photoluminescence fits

SI8. Estimation of efficiency

SI1. Experimental and band structure calculation methods, and shell thickness estimation

The growth procedure is illustrated in Figure S1 and further information on nanowire (NW) size optimization and alloy calibration are reported in [1]. The growth sequence starts with the growth of the GaAs NW stem at 560 °C for 25 min using fixed triethylgallium (TEGa) and tert-butylarsine (TBAs) precursors. A 5 min ramp-up interval followed, to reach the optimal conditions for GaP growth (610 °C) and adjust pressures for TEGa and tert-butylphosphine (TBP). Next the GaP segment was grown for 15 min. Following this, a growth interruption (GI) was employed to stabilize the TBAs and TBP line pressures for $GaAs_xP_{1-x}$ QD growth with 70% or 90% As, which lasted 25 s. Lastly, the GaP tip was grown for 15 min, during which a thin radial deposition occurs resulting in the few-nanometer-thick passivation shell. STEM observations showed that the passivation shell has nearly constant thickness on all wires, regardless of their diameter, as can be seen in Figure S2.

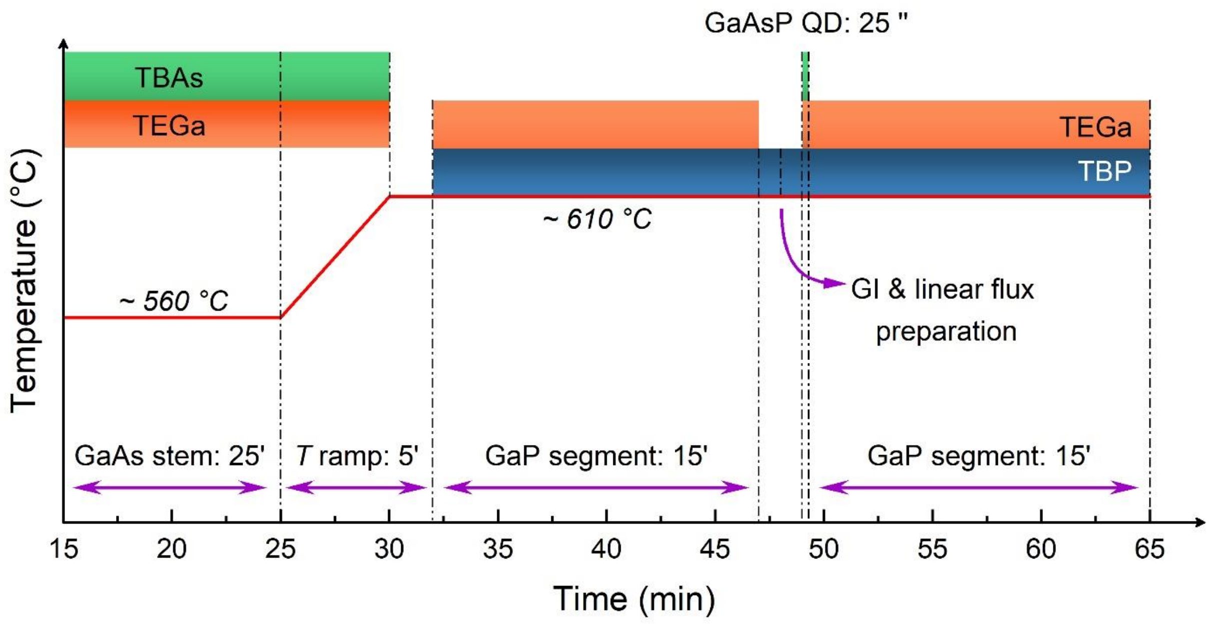

Figure S1. Schematics of the growth protocol used to grow the NW QD heterostructure. The growth temperature is shown by the red line. At the end of the GaAs stem growth, a short

temperature ramp toward the GaP growth temperature can be seen. The GI before the QD serves the purpose of stabilizing the precursors' partial line pressures and ensuring the formation of sharp interfaces.

Post-growth NW characterization imaging was performed via scanning electron microscopy (SEM; Zeiss Merlin operated at 5 kV). To comprehensively assess the NWs' morphology and dimensions, 45° side-view orientation of the as-grown samples was captured. Transmission electron microscopy (TEM), scanning transmission electron microscopy (STEM), energy dispersive X-ray (EDX) spectroscopy, and three-dimensional electron diffraction (3DED) were conducted using a JEOL JEM-F200 Multipurpose microscope, working at 200 kV and equipped with a Schottky field-emission gun and an EDX silicon-drift detector. TEM images were recorded with a GATAN RIO16 CMOS camera. 3DED data were recorded with an ASI CHEETAH hybrid-pixel detector and analyzed by the software PETS2[2].

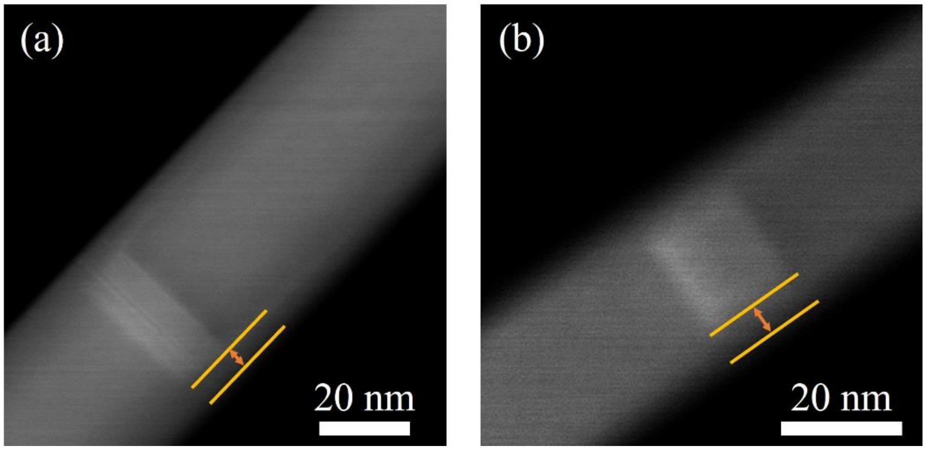

Figure S2. STEM images of representative $GaAs_{0.7}P_{0.3}$ QDs embedded in a thick and long (a) and thin and short (b) GaP NW. Both NWs show a nearly constant shell thickness regardless of their diameter, see lines in yellow.

Temperature-dependent microphotoluminescence (μ-PL) measurements were performed by utilizing a closed-cycle helium cryostat operating at controlled temperature, in which the samples were placed on a piezoelectric stage. As continuous-wave (CW) excitation source, a 532 nm solid-state laser was used with controlled excitation power, selected to avoid damaging the NWs. The light was focused through a 100× objective with a numerical aperture of 0.75, resulting in a diffraction-limited spot size of ~750 nm. The signal was collected in a backscattering configuration and dispersed by a spectrometer with a focal length of 50 cm and equipped with a grating with groove density of 300 grooves/mm, providing a spectral resolution of approximately 0.4 meV. Spectra were detected by a liquid nitrogen-cooled silicon CCD detector. NW ensembles were measured on the as-grown GaAs substrate and thus μ-PL signal wavevector was parallel to the standing NW long axis.

Time-resolved PL (TRPL) and autocorrelation measurements were performed by exciting samples with a tunable supercontinuum light source with variable repetition rate (0.15 - 78 MHz). The light output was spectrally filtered to provide tunable emission from 450 nm to 1100 nm, excitation at 525 nm was chosen for autocorrelation measurements. To ensure precise synchronization between each excitation pulse and photon detection, before hitting the sample, 10% of the laser light is diverted to a diode providing electrical triggers. By using the same optical path of the PL measurements, the sample is excited, its signal collected and dispersed by the spectrometer. Instead of being fed to the CCD, the sample's emission is sent by a mirror towards an exit slit and a focusing lens. In TRPL experiments the signal is focused on a micro-photon device (MPD) single-photon avalanche photodiode, which detects arrival of photons in the visible-range of light with high efficiency (up to 45% at 650 nm) and good timing resolution

(35 ps). The photodiode trigger signal and the single-photon event signal are fed into a time tagger that records time delays with ps precision.

In autocorrelation measurements, both with pulsed and CW excitation, the dispersed signal of the sample passes through a 50:50 beam-splitter and each beam is focused on 1 single-photon counting module (SPCM) with 350 ps timing resolution and up to 70% detection efficiency at 650 nm. Coincidence counts are then sent to the time tagger that measures the temporal correlation. To ensure comparable excitation conditions in CW and pulsed, the optical power used in the pulsed-laser measurements was adjusted to match the effective excitation conditions under CW excitation while also avoiding excessive power that could damage the emitter. Considering the laser repetition rate (~80 MHz) and the pulse duration (~30 ps), we estimated an empirical correction factor of P_{CW}/P_{pulsed} ~2.4 between the two excitation schemes. After applying this correction, the sample showed comparable emission intensity in both CW and pulsed experiments.

Electronic band structure and energy levels of $GaAs_xP_{1-x}$ were calculated through Nextnano software[3] by self-consistently solving 3D effective-mass Schrödinger and Poisson equations at 5 K. Numerical calculations involved simulating in a 3D environment a single wurtzite (WZ) GaP NW with embedded WZ $GaAs_xP_{1-x}$ QD with varying diameter, height and incorporated As% with imposed hexagonal symmetry. The band structure and material parameters of bulk WZ $GaAs_xP_{1-x}$ alloy were calculated by linear interpolation between the band structures of WZ GaP and WZ GaAs, derived by density functional theory calculations (full details are reported in [4]). High-symmetry point effective masses were estimated through parabolic fitting of WZ GaP and WZ GaAs band dispersions strictly near the high-symmetry points. Lattice parameters were measured by 3DED in TEM on reference samples of WZ GaP and WZ GaAs NWs with low

defect density and negligible strain, obtaining respectively a = 3.89 Å, c = 6.40 Å and a = 4.01 Å, c = 6.64 Å. $GaAs_xP_{1-x}$ lattice constants were obtained by linear interpolation between the parameters of the two binary alloys. Dielectric constants calculated in [5] were used.

SI2. Additional optical spectroscopy data on multiple quantum dots

Figure S3a shows multiple μ-PL spectra at 5 K of wurtzite GaP NW with embedded $GaAs_{0.7}P_{0.3}$ QDs, acquired from different points of the as-grown ensemble. The spectra are characterized by multiple narrow peaks that can be associated with the different standing NW QDs illuminated in this configuration by the laser spot. The differences in energy and *fwhm* are due to the variability in the sizes of the different QDs, both diameter and height. As discussed in the main text, the size determines the degree of quantum confinement of the system and, consequently, the observed recombination energy: it is possible to observe intense, QD-like emission with energy ranging from ~1.80 eV (larger confinement volume) to ~1.95 eV (smaller confinement volume). This phenomenon affects the tunability of the emission achieved through the controlled incorporation of As in the QD during growth. For this reason, it is possible to find $GaAs_{0.7}P_{0.3}$ QDs emitting in the same range of $GaAs_{0.9}P_{0.1}$ QDs, see Figure S3b.

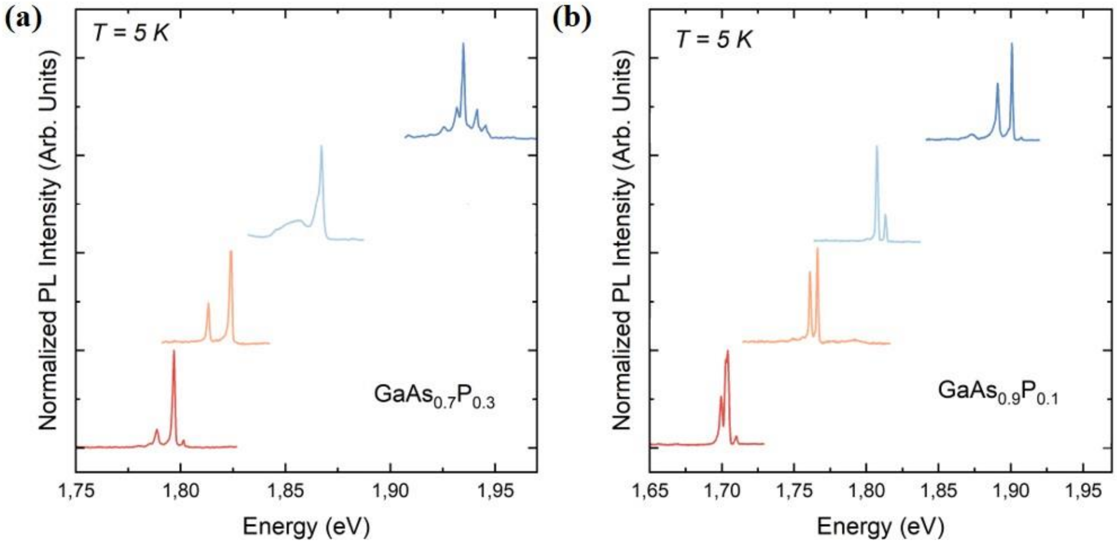

Figure S3. μ-PL spectra, at 5 K and P = 1 μW, of wurtzite GaP NW with embedded $GaAs_{0.7}P_{0.3}$ (a) and $GaAs_{0.9}P_{0.1}$ (b) QDs, acquired on different positions of the as-grown ensemble. Data shows how the great variability of confinement volume existing in each sample limits the emission tunability that would be achieved through mere control of As incorporation in the QD. In each spectrum, multiple excitonic lines can be seen, associated with light collected from neighboring QDs excited by the laser spot. The most intense and narrow line was chosen for the μ-PL characterization and autocorrelation measurements reported in the main text.

SI3. Confinement as a function of diameter and height

Figure S4 shows the scatter plot of calculated $GaAs_{0.7}P_{0.3}$ QD emission energy as a function of both radius (*r*) and height (*h*), giving rise to the QD volume values in Figure 2 in the main text. Calculations were performed with Nextnano software choosing QD *r* and *h* to match the statistics obtained through STEM measurements. Two populations of QDs are observed, which can be divided into $r > 8$ nm and $r \leq 8$ nm (see Fig. 2a of the main text), and calculated: with $r > 8$ nm and $h > 13$ nm, $r < 8$ nm and $h < 8$ nm. In larger QDs, quantum confinement is weak, confirmed by the emission energy of ~1.70 eV, just above the bandgap of WZ $GaAs_{0.7}P_{0.3}$. In smaller QDs, the energy emission increases, consistent with greater confinement. Notice that quantum confinement is also affected by carrier effective masses anisotropy along different crystallographic directions, as typical of wurtzite crystals, and this contribution was properly taken in to account in the calculations. Overall, the contribution of *r* and *h* to the confinement is slightly different, as can be better appreciated in Figure S5, where calculations over an extended range of height and diameter combinations are reported. QD emission energy is plotted as a function of the QD diameter with fixed height, each color represents QDs with the same height. The blue stars approximately represent the QDs measured by STEM and restrict the possible height and diameter combinations. Thanks to this double correspondence, it was possible to estimate the QD volume associated with each experimentally measured QD transition energy (orange triangles in Figure 2b of the main text). The choice to use volume was made considering that, given the partial degeneracy between different height/diameter combinations yielding similar transition energies, a volume-based representation provides a more complete and conservative comparison between simulations and experimental data, without implying that confinement is strictly isotropic.

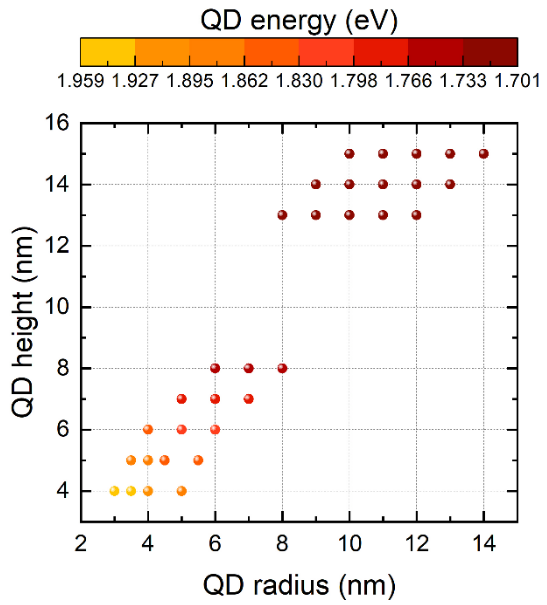

Figure S4. Color mapped scatter plot of $GaAs_{0.7}P_{0.3}$ QD emission energy as a function of both radius and height. Points were obtained by performing calculations with Nextnano software and choosing dimensions consistent with HR-STEM measurements of QD dimensions.

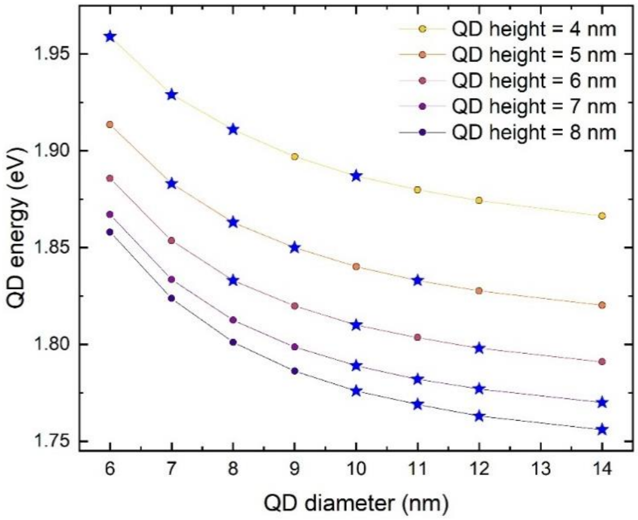

Figure S5. Plot of the QD emission energy as a function of the QD diameter with fixed height, calculated with nextnano software. Each color represents QDs with same height. Blue stars highlight STEM measured QDs, which loosely follow Gibbs-Thomson distribution.

SI4. Band edge diagrams of QDs of different size and As%

Figures S6a and S6b show the calculated band edge diagrams of a $GaAs_{0.7}P_{0.3}$ QD with different dimensions (QD diameter = 8 nm and QD height = 5 nm in S6a, QD diameter = 14 nm and QD height = 8 nm in S6b), embedded in a WZ GaP NW. The energy of the first transition changes from 1.863 eV to 1.756 eV, as the QD volume is increased, showing a direct dependence on the confinement volume: the system is less confined and the discrete levels are closer to each other. Figure S6c shows a $GaAs_{0.9}P_{0.1}$ QD simulated with the same diameter, 7 nm, and height, 4 nm, as the one with 70% As whose band edge diagram is reported in the main text. The emission energy changes from 1.923 (70%) eV to 1.827 eV (90 As%). It can be thus observed that, for equal dimensions, the tunability achieved through controlled As incorporation in the QD is retained, as expected.

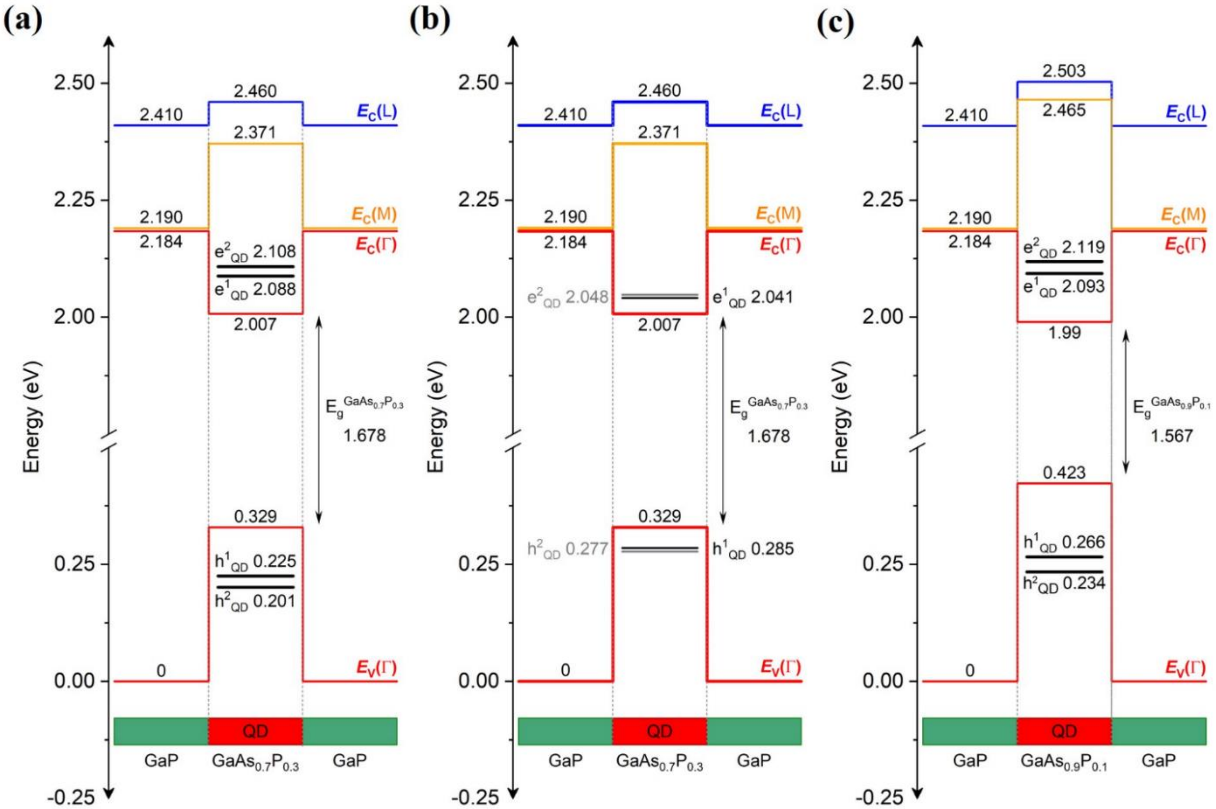

Figure S6. Band edge diagrams, calculated with Nextnano software, of the NW QD heterostructure showing the WZ GaP barriers and a $GaAs_xP_{1-x}$ QD. The zero-energy level energy is set at the valence-band maximum of WZ GaP (a) QD with 70% As with diameter of 8 nm and height of 5 nm. QD first transition recombination energy is 1.863 eV. (b) QD with 70% As with diameter of 14 nm and height of 8 nm. QD first transition recombination energy is 1.756 eV. (c) QD with 90% As with diameter of 7 nm and height of 4 nm. QD first transition recombination energy is 1.827 eV. All given numbers in the diagram are energies in eV.

SI5. Power- and temperature-dependent optical characterization of QD_2 and QD_3

Figures S7a and S7c show integrated μ-PL intensity as a function of impinging power, of the peaks QD_2, 1.932 eV, and QD_3, 1.926 eV, associated with QD located near QD_1 emitter and thus also excited by our laser spot. Optical characterization was performed while maximizing signal of QD_1, so that the power densities reaching QD_2 and QD_3 are greatly overestimated. As shown in the figure insets, at low power intensity increases linearly for both lines, $I_{det} \propto (P_{exc})^M$ with $M_2 = 1.12 \pm 0.02$ and $M_3 = 0.96 \pm 0.06$, as characteristic of single excitonic transitions. At higher powers, intensity saturation occurs with a sublinear trend. Figures S7b and S7d show temperature-dependent integrated PL intensity of QD_2 and QD_3, respectively. Red lines show the fitting by an Arrhenius-like model with the extracted activation energies. Quantum dots formed in nearby nanowires are expected to experience very similar local growth conditions (e.g., precursor flux, temperature, and local geometry), which can lead to correlated structural parameters such as dot size and composition. As a result, emitters located in close spatial proximity can exhibit comparable emission energies but variations in local strain or electrostatic environment will contribute to the precise value observed, hence the different energies for different emitters. Optical characterization indeed shows that QD_2 and QD_3 peaks arise from single exciton recombination and not from double or bound exciton of QD_1.

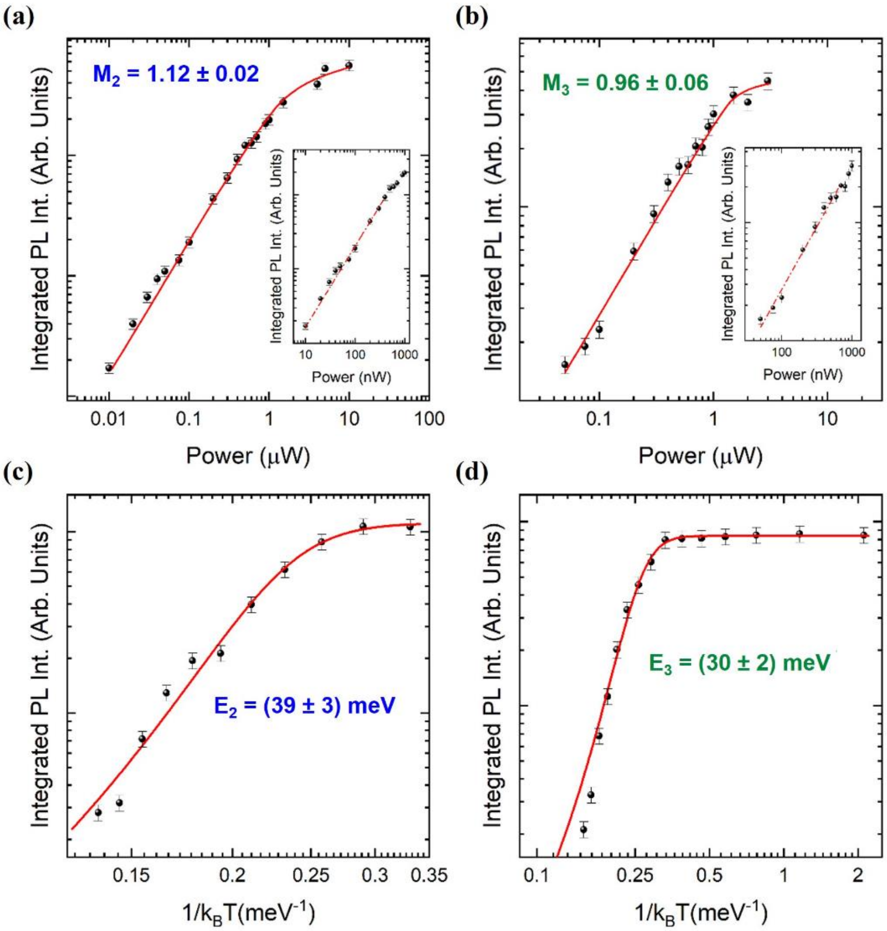

Figure S7. Power- and temperature-dependent μ-PL measurements on $GaAs_xP_{1-x}$ QD NWs with As = 70%. (a) – (b) Plot of the power-dependent integrated intensity of QD_2 and QD_3, respectively. The red line shows the fit with $I = I_{sat}\left[\frac{P}{P+P_N}\right]$, where P_N is the laser power at which the intensity is half of I_{sat}. The fits yield P_N = 2.6 ± 0.2 μW for QD_2 and P_N = 1.7 ± 0.3 μW for QD_3. The insets are a zoom below P_0 (defined in the main text) to highlight the linear trend of the exciton before saturation. The red dashed line shows the fit with $I_{det} \propto (P_{exc})^M$ that yields M_2 = 1.12 ± 0.02 and M_3 = 0.96 ± 0.06. (c) – (d) Plot of the temperature-dependent integrated intensity of QD_2 and QD_3, respectively, as a function of the reciprocal of the temperature with the Arrhenius fit (red line) and extracted activation energy. QD_2 was not

measured at low temperatures since during the temperature study the laser position was optimized on the QD_1 line.

SI6. Dot emission spatial localization

Figure S8a and S8b show 5 K μ-PL maps made with 200 nm steps along the X and Y sample axes, of the as-grown ensemble of wurtzite GaP NW with embedded $GaAs_{0.7}P_{0.3}$ QD. SI7a reports the intensity map of the QD_1 excitonic line, with a peak at 1.935 eV. SI7b instead reports the map related to QD_2, 1.932 eV. Due to the interplay of NW density on the substrate and Gaussian profile of the laser spot, spatial origin of each contribution can be assessed by moving the X and Y axes to maximize the intensity of the respective excitonic line by positioning directly over the NW. As excitation power increases, the "tails" of the spot carry enough power to excite nearby QDs and so QD_2 and QD_3 emission become visible. The QD from which the QD_1 peak originates is located near the coordinates (-0.1,0.1) μm of the map while QD_2 is in (0.6,0.1) μm. The uncertainty on the position of the NW QD comes from two contributions. The first is the size of the laser spot with which the QD is excited and the signal collected, which is slightly bigger than the distance between the two QDs. However, the spatial resolution is nearly half of the spot size, which explains why we can separately maximize the signals of the two emitters. The second contribution comes from the encoder error of our nanometric stage equal to ~50 nm along the X direction and ~100 nm along the Y direction, but this is negligible with respect to the spatial resolution. Despite all this, the maps in Figure S8 prove that it is possible to spatially resolve the different contributions to the emission and study the various QDs almost individually.

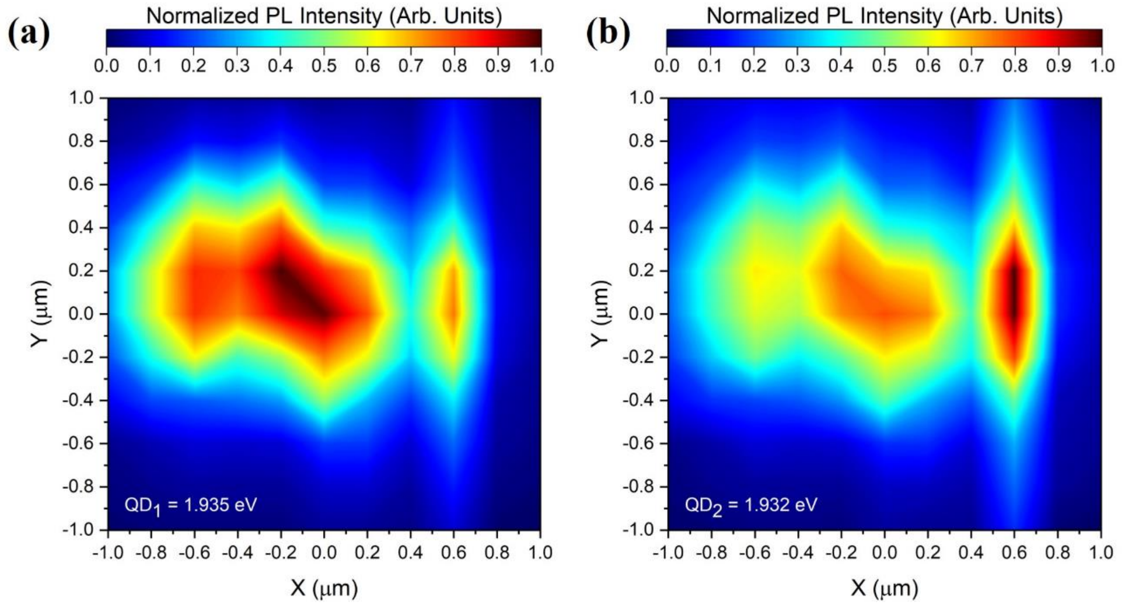

Figure S8. 5 K μ-PL maps made with 200 nm steps along the X and Y axes. (a) Normalized intensity map of the QD_1 excitonic line at 1.935 eV, whose NW QD results to be in position (-0.1,0.1) μm. (b) Normalized intensity map of the QD_2 excitonic line at 1.932 eV, whose NW QD results to be in position (-0.6,0.1) μm.

SI7. Power- and temperature dependent $g^{(2)}(0)$ and TRPL fits

Here are reported the fit results of power- and temperature dependent $g^{(2)}(0)$ measurements under CW excitation at 5 K showed in Figure 4a and 4b of the main text. In employing the simplified two-level rate equation[6]: $g^{(2)}(\tau) = 1 - \exp[-\tau\left(\frac{1}{\tau_1} + G\right)]$, τ_1 was independently measured from TRPL measurements performed under the same experimental conditions. Data were fitted with the convolution of exponential decay law e^{-t/τ_1} with the instrument response function (IRF), which takes into account temporal delays and distortions introduced by the experimental setup. Figure S9a shows 5 K TRPL measurements of the QD_1 spectral line excited at various powers (black dots), the red lines show the fit from which τ_1 was estimated at each power, and the blue line is a representative measured IRF function. By plugging the TRPL results in the two-level rate equation it was possible to fit the power-dependent CW $g^{(2)}(0)$ measurements and extract the CW photogeneration rate of e–h pairs (G), see Figure S9b. As expected, the effective photogeneration rate G increases with increasing excitation power. At sufficiently high pumping conditions, the increased carrier injection enhances the probability of re-excitation and multiphoton events, leading to the observed increase of $g^{(2)}(0)$ above 0.5, as shown in the main text. Figure S10 shows 40 K and 70 K TRPL measurements of the QD_1 spectral line (black dots), the red lines show the fit from which τ_1 was estimated at each temperature.

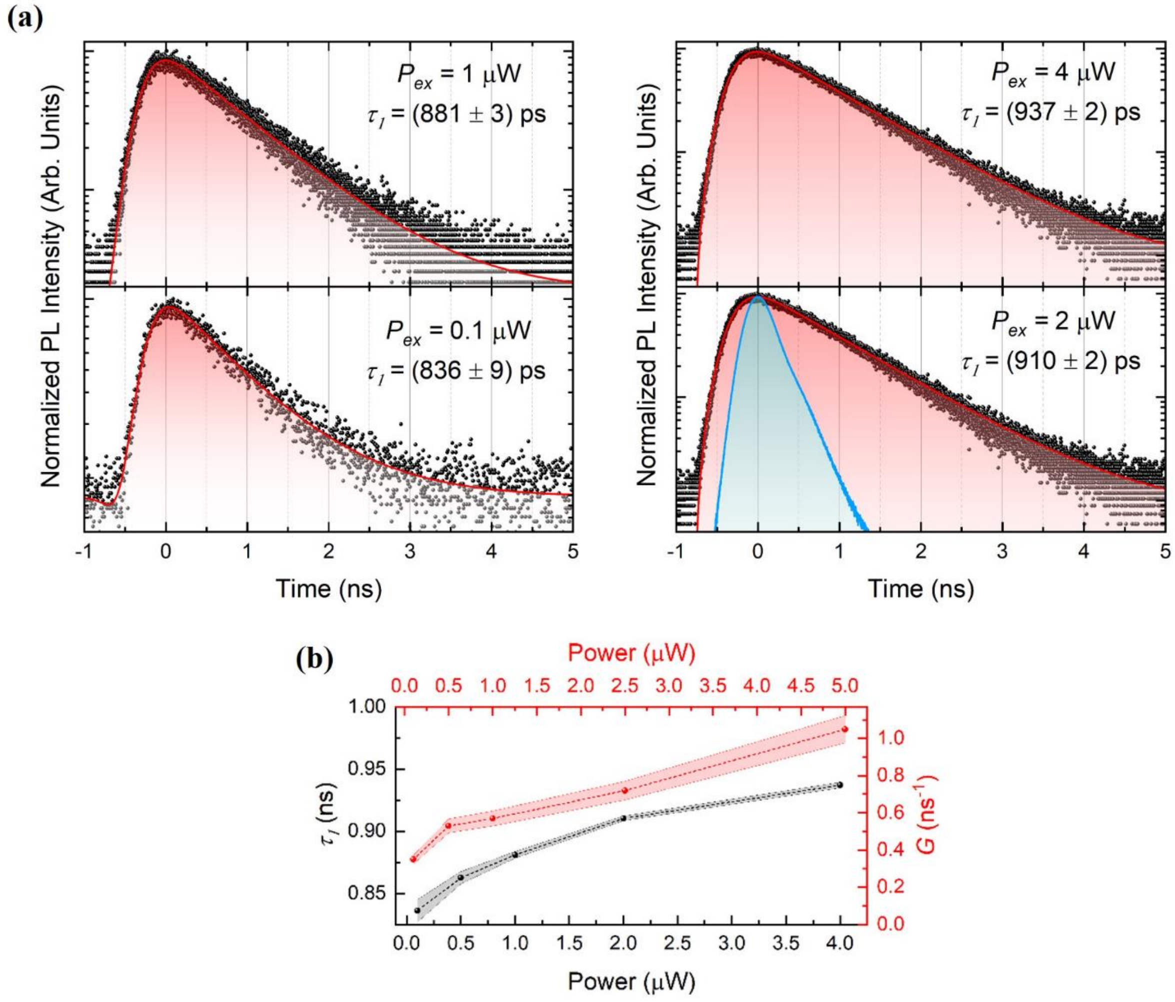

Figure S9: (a) Power-dependent time-resolved PL on the QD_1 spectral line at 5 K. Data were fitted (red lines), with a single-exponential decay function convoluted with the system response (blue curve). (b) Fit results showing the power-dependent trend of QD_1 radiative lifetime τ_1, black dots, and photogeneration rate of e–h pairs (G), red dots. The shaded area is included within the error associated with the reported quantities. Autocorrelation measurements from where G was extracted were performed with CW excitation, while τ_1 was measured with pulsed excitation. Power under pulsed excitation regime was experimentally adjusted to obtain comparable emission count rates from the QD with CW regime, and thus the reported values are

corrected by an empirical correction factor that is influenced by the repetition rate used and the pulse width of the laser (~30 ps). Correction factor is P_{CW}/P_{pulsed} ~2.4.

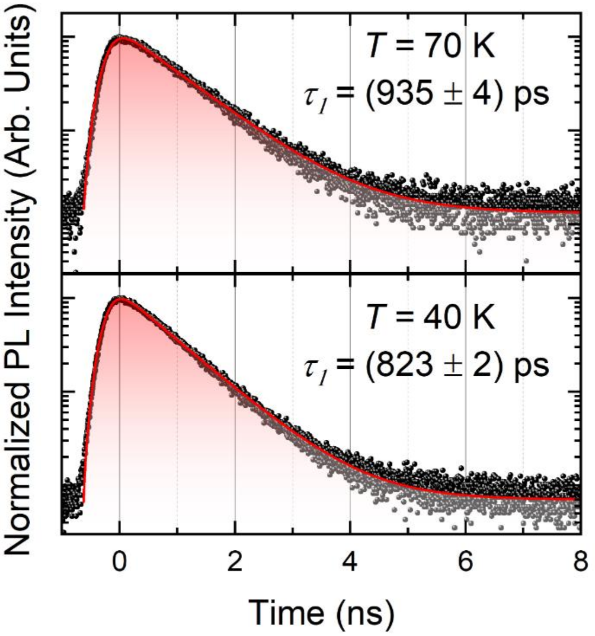

Figure S10: Temperature-dependent time-resolved PL on the QD_1 spectral line at 40 K and 70 K at P_0 = 1 μW excitation power. Data fitted (red lines) with a single-exponential decay function convoluted with the system response.

SI8. Estimation of efficiency

To estimate the intrinsic photon extraction efficiency of the QDs, it is necessary to measure the setup efficiency (η_{setup}) at the QD wavelength. We used a pulsed laser with the same wavelength as our QD and measured the effective transmission of each optical component in the setup, resulting in η_{setup} = 8.5% ± 1%. The QD extraction efficiency, η_{extrac} is given by [7]:

$$\eta_{extrac} = \frac{C}{\eta_{det} * \eta_{setup} * R_{laser}} \quad \text{(S1)}$$

where η_{det} is the APD detector efficiency at the QD emission wavelength (70% at 640 nm), R_{laser} is the repetition rate of the laser (~ 80 MHz at 640 nm excitation wavelength) and C are the detected counts/s on the APD detectors (~ 44 kcounts/s). The estimated efficiency is equal to 0.9% ± 0.2%. This quite low value is however very promising, because these thin NWs do not support waveguiding effects which can be achieved via growth of thicker nanowires (~ 170 nm) or the use of an external cavity.

REFERENCES

(1) Sorodoc, R. A.; De Vincenzi, P.; Sharma, A. S.; Bucci, G.; Roggi, M.; Mugnaioli, E.; Sorba, L.; De Luca, M.; Zannier, V. Tunable $GaAs_{x}P_{1-x}$ Quantum-Dot Emission in Wurtzite GaP Nanowires. *ACS Appl. Mater. Interfaces* **2024**, *16* (47), 65222–65232. https://doi.org/10.1021/acsami.4c15343.

(2) Palatinus, L.; Brázda, P.; Jelínek, M.; Hrdá, J.; Steciuk, G.; Klementová, M. Specifics of the Data Processing of Precession Electron Diffraction Tomography Data and Their Implementation in the Program *PETS2.0*. *Acta Crystallogr. Sect. B Struct. Sci. Cryst. Eng. Mater.* **2019**, *75* (4), 512–522. https://doi.org/10.1107/S2052520619007534.

(3) Birner, S.; Zibold, T.; Andlauer, T.; Kubis, T.; Sabathil, M.; Trellakis, A.; Vogl, P. Nextnano: General Purpose 3-D Simulations. *IEEE Trans. Electron Devices* **2007**, *54* (9), 2137–2142. https://doi.org/10.1109/TED.2007.902871.

(4) Giorgi, G.; Amato, M.; Ossicini, S.; Cartoixà, X.; Canadell, E.; Rurali, R. Doping of III–V Arsenide and Phosphide Wurtzite Semiconductors. *J. Phys. Chem. C* **2020**, *124* (49), 27203–27212. https://doi.org/10.1021/acs.jpcc.0c09391.

(5) De, A.; Pryor, C. E. Optical Dielectric Functions of Wurtzite III-V Semiconductors. *Phys. Rev. B* **2012**, *85* (12), 125201. https://doi.org/10.1103/PhysRevB.85.125201.

(6) Regelman, D.; Mizrahi, U.; Gershoni, D.; Ehrenfreund, E.; Schoenfeld, W.; Petroff, P. Semiconductor Quantum Dot: A Quantum Light Source of Multicolor Photons with Tunable Statistics. *Phys. Rev. Lett.* **2001**, *87* (25), 257401. https://doi.org/10.1103/PhysRevLett.87.257401.

(7) Denis, N.; Sharma, A. S.; Dede, D.; Nurmamytov, T.; Cianci, S.; Santangeli, F.; Felici, M.; Boureau, V.; Polimeni, A.; Rubini, S.; Fontcuberta I Morral, A.; De Luca, M. Single Photon Emitters in Thin GaAsN Nanowire Tubes Grown on Si. *ACS Nano* ***2025**, 19 (46), 39757–39767.* https://doi.org/10.1021/acsnano.5c12139.
